# Parents’ perspectives of anorexia nervosa treatment in adolescents: a systematic review and metasynthesis of qualitative data

**DOI:** 10.1186/s40337-023-00910-z

**Published:** 2023-10-30

**Authors:** Ngozi O. Oketah, Jacqueline O. Hur, Jonanne Talebloo, Chloe M. Cheng, Jason M. Nagata

**Affiliations:** 1 Department of Paediatrics, Children’s Health Ireland (CHI) at Crumlin & Connolly Hospitals, Cooley Road, Dublin, D12 N512 Ireland; 2 Division of Adolescent and Young Adult Medicine, Department of Paediatrics, University of California San Francisco, 550 16th Street, 4th Floor, Box 0503, San Francisco, CA 94143 USA

**Keywords:** Anorexia nervosa, Adolescent, Parents, Qualitative, Systematic review, Metasynthesis

## Abstract

**Background:**

Studies have established the central role of the family in the recognition, treatment, and recovery of anorexia nervosa. The objective of this study was to review, synthesize, and critically appraise the literature on parents’ views on the treatment and recovery process of anorexia nervosa in their adolescent child.

**Method:**

A systematic search of Medline, PsychINFO, CINHAL, EMBASE, Cochrane library, and SSCI was conducted for qualitative studies published regarding parents’ views about the treatment of anorexia nervosa. The quality of articles was assessed using the critical appraisal skills program (CASP) and findings were analysed using thematic synthesis.

**Results:**

A total of 25 studies from nine countries reporting the views of 357 parents met the inclusion criteria. Four major themes were developed from the analysis: understanding the child and the disease, experience of services and treatment modalities, the role of professionals, and the experience of recovery.

**Conclusion:**

Parents report struggles with delays in finding help, judgmental attitudes of professionals, and uncertainty about the future. Recognition of the challenges faced by parents and families empowers clinicians to build stronger therapeutic relationships essential for long-term recovery from anorexia nervosa.

**Supplementary Information:**

The online version contains supplementary material available at 10.1186/s40337-023-00910-z.

## Abstract (plain language summary)

Interventions for adolescents with anorexia nervosa often focus on families. Although prior systematic reviews have incorporated adolescents’ perspectives on their treatment for anorexia nervosa, none have previously reviewed parents’ perspectives on their adolescents’ treatment. We conducted a systematic review and metasynthesis of qualitative data capturing parents’ perspectives of anorexia nervosa treatment in adolescents. Four themes emerged from the analysis: understanding the child and the disease, experience of services and treatment modalities, the role of professionals, and the experience of recovery. Parents report struggles with delays in finding help, judgmental attitudes of professionals, and uncertainty about the future. Recognition of the challenges faced by parents and families empowers clinicians to build stronger therapeutic relationships essential for long-term recovery from anorexia nervosa.

**Supplementary Information:**

The online version contains supplementary material available at 10.1186/s40337-023-00910-z.

## Introduction

Anorexia nervosa (AN) is a severe eating disorder (ED), with its onset typically occurring in adolescence and with it being more common in females. It stands out as not only a severe mental illness but also as a severe chronic disease, as it has the highest mortality rate of any psychiatric illness [1, 2]. AN presents with acute and chronic morbidities that affect every organ system of the body, which increases the risk of mortality and long-term chronic health problems [3]. Additionally, people with AN show a marked disturbance in cognitive and emotional functioning.

Most interventions studied to date for adolescents with AN focus on the families [3], which largely depend on the support and commitment of the caregivers to effect a behavioural change for the recovery of the young person. Research has shown the efficacy of family-based treatment (FBT) in the treatment of AN [4]— a modality where parents and siblings play a central role in the recovery of the child.

Although quantitative studies on EDs help us evaluate outcomes of various treatment options, qualitative studies improve our understanding, thus giving us insight into and a description of the complex issues involved in treatment from the perspective of the participants [5]. Qualitative research is intended to highlight the deeper significance that the subject of the research ascribes to the topic being researched and involves an interpretive, naturalistic approach, giving priority to what the data contribute to important research questions or existing information [6].

Three systematic reviews have been published on adolescents’ views of their treatment for AN. Espindola and Blay [7] investigated perspectives from both adults and adolescents, while the review from Bezance and Holliday [8] solely focused on adolescents. Westwood and Kendal [9] published a review of literature that included both qualitative and quantitative research, although they used a thematic synthesis to describe their findings. All three studies were united in emphasizing the importance of parents in their adolescents’ care but did not explicitly include the parents’ perspectives.

Two metasyntheses were published by Sibeoni et al. in 2017, looking at the intersection of perspectives from adolescents, parents, and health professionals: one on the lived experience [10] and the other on views about the treatment of AN in adolescents [5]. The former metasynthesis found that adolescents were primarily concerned about the psychological and emotional impact of having AN, while parents and health professionals were primarily concerned about patients’ physical bodies, indicating a need for integrated perspectives when seeking to effectively treat AN; the study also focused mainly on parental perspectives of their adolescents’ experience with AN, rather than on AN treatment. The latter metasynthesis found that adolescents, parents, and health professionals differed in their treatment targets, with adolescents focusing on the present, parents focusing on family history and the past, and providers focusing on risks for the future. The study findings and discussion mainly centered on the differing perspectives of adolescents and health professionals, which was found to pose a barrier to the development of a positive therapeutic alliance, rather than on parental perspectives.

To date, no published systematic review focuses solely on parents regarding the treatment their child has received for AN. Given the importance of parental involvement in the treatment and recovery process of AN in adolescents, we sought to explore the experience of treatment for adolescents with AN from a parental perspective with some perspective on the care and recovery process during treatment using a systematic review of qualitative studies.

## Methods

We conducted a systematic review of qualitative studies exploring parents’ experiences and perspectives on the treatment and recovery process of AN in adolescents.

Qualitative research provides evidence for the development, implementation, and evaluation of health interventions [11] and remains an important mechanism for improving knowledge about the treatment of adolescents.

Metasynthesis, or qualitative evidence synthesis, is a systematic review and integration of findings from individual qualitative studies to create new understanding by comparing and analysing concepts and findings from different sources of evidence, focusing on the same topic of interest. We used a ‘thematic synthesis’ approach to metasynthesis [11, 12].

Thematic synthesis involves three stages: the coding of text 'line-by-line'; the development of 'descriptive themes' which remain close to the primary studies; and the generation of 'analytical themes,' where the authors generate new interpretive constructs or hypotheses [13].

### Search strategy

We conducted a systematic search of six databases: Medline, PsychINFO, CINHAL, Embase, Cochrane Library, and Web of Science (Table 1). The keywords were determined using existing literature and included: anorexia, anorexia nervosa, restrictive eating disorder, treatment, therapy, experience, recovery, parent, guardian, carer, qualitative, interviews, narratives, views, perceptions, and knowledge, combined with the Boolean logic terms “and” and “or.” The reference sections of the potentially relevant papers were examined, as were other articles that cited these papers, in order to further gather relevant studies.

**Table 1:** Results of search strategy for databases

Database	Dates	Result
PsycINFO (EBSCO publishing) 1800	01/01/1980 to 08/01/2023	121
Embase (Ovid) 1974	01/01/1980 to 08/01/2023	75
Medline (Pubmed) 1948	01/01/1980 to 08/01/2023	112
CINAHL plus (EBSCO publishing) 1981	01/01/1980 to 08/01/2023	88
Web of science 1970	01/01/1980 to 08/01/2023	175
Cochrane library 1944	01/01/1980 to 08/01/2023	22

### Selection criteria

Identified studies from these databases were individually evaluated and selected based on inclusion and exclusion criteria agreed upon by the authors.

Studies were included if they used a qualitative methodology for data collection and analysis, were published in the English language between January 1980 and August 1, 2023, focused on treatment or recovery (which occurs during a treatment process) of AN during adolescence, and had parents as participants.

Studies were excluded if they used quantitative or mixed methodology, if AN was not the primary diagnosis, if non-specific EDs were discussed rather than AN, and if they did not focus on treatment or recovery.

### Assessment of article quality

The authors critically assessed the quality of the articles selected to judge the integrity of their data using the Critical Appraisal Skills Program (CASP) [14, 15]. The CASP has previously been used in other qualitative metasyntheses [5, 16, 17]. Three authors initially rated studies for quality (NO, JH, JT), with discrepancies resolved after discussion with all authors (including CC and JN).

The CASP consists of ten questions and provides a system for appraising the quality of each qualitative research article by considering how well the authors present steps taken during their study and the rationale behind each step.

The ten questions comprise two screening questions about the aims of the research and the appropriate use of a qualitative methodology, and the other eight questions ask about the research design, data collection, and data analysis process, sampling strategy, management of ethical issues, and the value and presentation of the findings of the research.

### Data analysis

Thematic analysis was used to extract key themes from the chosen studies, following the approach used in much qualitative research [18]. Papers identified from the literature search were independently and systematically read line by line and reviewed with note-taking by three coauthors (NO, JH, JT). Concepts and representative quotations were first extracted from the results of each study. After thorough individual reviews, all coauthors met to create a concept map of these themes, which enabled us to visualize connections and identify thematic saturation. As subsequent papers were identified to account for recent publications, new themes were identified, or old themes were modified.

Reciprocal translation was used to compare themes across papers to ensure that each theme encompassed similar concepts within each study [13]. This process yielded four central themes and twelve sub-themes that were agreed upon by all authors.

### Reflexivity

The research team has reflected on their positionality related to the topic and thematic analysis, as it is important to identify and reflect on any preconceptions that could influence the research process. NO identifies as an African Irish cisgender female. She is a pediatrician with an interest in adolescent physical and mental health and provides medical treatment for adolescents with AN. JH identifies as an Asian American cisgender female. She is studying psychology and public policy and has research interests in mental health and EDs. JT identifies as an Iranian American cisgender female. She is studying molecular and cell biology and has research interests in adolescent mental health and EDs. CC identifies as an Asian American cisgender female. She is a medical trainee with clinical and research interests in mental health and EDs. JN identifies as an Asian American cisgender male. He is a pediatrician specializing in medical management of EDs who provides medical treatment for adolescents with AN and their families. These experiences could influence the authors’ preconceptions of EDs and related treatment. The analysis by multiple authors, discussion of discrepancies, and reciprocal translation was meant to minimize the impact of these preconceptions on the extraction and meta-synthesis.

## Results

### Overview of studies

**Fig. 1:**
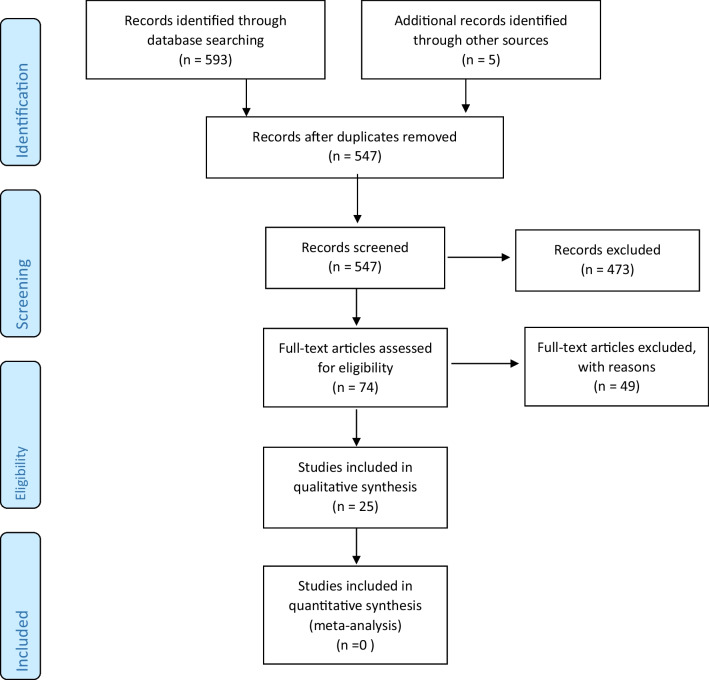
Different phases of study selection PRISMA 2009 flow diagram

We followed PRISMA guidelines, which are shown in Fig. 1. In total, the systematic search yielded 598 papers, of which 547 were unique. 473 articles were eliminated after reading the titles, as the titles indicated exclusion criteria were met. After reading 74 articles, 25 met the inclusion criteria. Studies were excluded because the methodology was not qualitative, participants did not predominantly have AN, the subject was not focused on treatment or recovery, and the perspective of treatment did not include carers or parents of adolescents. Of note, one study from the USA [19]—the first qualitative study addressing the parenting needs of adolescents in the treatment of ED—could not be included in the analysis, as it did not specify what ED the adolescents were diagnosed with.

Two of the included studies used ‘eating disorders’ in their title rather than AN but on reading the articles, we found that the parents interviewed (N = 26) all had children with AN. Two studies clearly stated the use of DSM-IV [20, 21], with one study the use of ICD-10 [22] criteria for diagnosis of their participants’ children, while the other studies only stated that adolescents were treated for AN.

### Participants

All studies included parents of adolescents either currently undergoing AN treatment or who had undergone treatment. Eight studies included responses from both parents, three studies from mothers only, twelve studies from parents and adolescents, and two studies from parents, adolescents, and siblings. Apart from two Chinese studies, most studies described their participants as white or British. No study specifically mentioned having an ethnic minority.

357 parents were interviewed, with mothers accounting for the majority of participants (203); a majority of their children were adolescent females.

### Setting

Seven studies took place in an outpatient setting, six in an inpatient setting, one in a home setting, and seven in mixed settings, which included a combination of inpatient, outpatient, and psychotherapeutic treatments. Four other studies stated that participants had completed treatment.

The extent of parental involvement in treatment varied between studies. Nine studies directly involved parents through FBT/Multi-Family Treatment (MFT), one study involved parents through a Family Admissions Program (FAP), three studies involved parents throughout the inpatient treatment process, and two studies involved parents through home treatment. Other included studies did not examine parents’ direct involvement in the treatment process but rather their perceptions of their children’s individual treatment.

Studies came from nine countries: seven from the United Kingdom, two from Ireland, two from China, two from Canada, three from France, two from Norway, five from Australia, and one each from the United States and Sweden.

The main characteristics of included studies are described in Table 2.

**Table 2:** Characteristics of chosen studies

Year	Authors	Title/aim	Parent and child characteristics	Data collection	Methodology/analysis	Country
1999	Sharkey-Orgnero [20]	Qualitative analysis of parents’ views of their daughters’ recovery from AN	18 Parents (10 mothers and 8 fathers) of females (aged 12–18 years)	Semi-structured interviews	Grounded theory	Canada
2003	Tan et al. [22]	To explore the views of patients and parents regarding compulsory treatment in anorexia nervosa	9 Parents (8 mothers and 1 father) of females (aged 13–21)	Semi-structured interviews	Grounded theory	United Kingdom
2004	Cottee-Lane et al. [23]	To describe the experience of parents having a child with AN	11 Parents (7 mothers and 4 fathers) of females (age 13–16)	Semi-structured interviews	IPA	United Kingdom
2005	Tierney [21]	To explore parents’ views in relation to treatment received by their children with AN	14 Parents (8 mothers and 6 fathers) of adolescents	Semi-structured interviews	Content analysis and coding (Atlas-Ti)	United Kingdom
2007	Honey et al. [24]	To address the question “What support do parents of teenage girls with anorexia nervosa want from clinicians?”	24 Parents (16 mothers and 8 fathers) of females (aged 14–20)	In-depth interviews	Content analysis	Australia
2008	Ma [25]	To explore the subjective experiences and effectiveness of family therapy by patients and families	Families of 18 females (including mothers and fathers) who had completed treatment	In-depth interviews	Content analysis	China
2014	Bezance and Holliday [26]	To explore mothers’ experience of home treatment (HT) for their daughters with AN	9 Mothers of adolescents a ged 13–16 years	Semi-structured interviews	IPA	United Kingdom
2015	McCormack and McCann [27]	To investigate the subjective experiences of parents caring for an adolescent with AN (including experience of services)	10 Parents (7 mothers and 3 fathers) of adolescents	Semi-structured interviews	Thematic analysis + NVivo 9	Ireland
2016	Engman-Bredvik et al. [28]	To investigate the experience of multi-family therapy as part of AN treatment from a parental perspective	12 Parents (6 fathers and 6 mothers) of 6 females (aged 12–17)	Semi-structured interviews	Empirical phenomenological psychological method (EPP)	Sweden
2017	Fink et al. [29]	To investigate patient and families experience in a family admissions program–for families at risk of unsuccessful outcomes with FBT	26 Parents (14 mothers and 12 fathers) of adolescents (aged 12–16)	Semi-structured interviews	Narrative Inquiry and IPA	Australia
2019	Mitrofan et al. [30]	To explore patient and parent perspectives on positive and negative aspects of care for YP with eating disorders	11 Parents–9 mothers and 2 fathers (9 daughters and 2 sons (aged 11–17 at diagnosis)	Focus groups	Thematic analysis + NVivo 9	United Kingdom
2019	McArdle [31]	To gain a more in-depth understanding of parents’ experiences of health services for the treatment of eating disorders	15 Parents (3 fathers and 12 mothers) of 12 females (aged 11–21)	Focus groups and semi-structured interviews	Interpretive thematic analysis	Ireland
2019	Medway et al. [32]	To explore perspectives of young people and parents regarding developmental impact of AN and the role of FBT in facilitating return to health adolescent development	12 Parents–10 mothers and 2 fathers of 11 females and 1 male (age of onset 12–16 years)	Semi-structured interviews	Narrative inquiry	Australia
2019	Wufong et al. [33]	To explore parents’ experiences of MFT/FBT where treatment was discontinued and/or their child continued to experience psychological distress post treatment for AN	13 Parents–9 mothers and 4 fathers of 11 female (aged 12–17 at diagnosis)	Semi-structured interviews	Critical discursive analysis	Australia
2021	Baumas et al. [34]	To explore patient and parent perceptions of multi-family therapy's effectiveness and underlying mechanisms	6 Parents (4 mothers and 2 fathers) of 3 adolescents (2 daughters and 1 son aged 14–19)	Focus groups	Thematic interpretation	France
2023	Baudinet et al. [35]	To explore adolescent and parent perceptions of changes occurring during MFT	15 Parents (10 mothers and 5 fathers) of 8 adolescents (aged 14–17)	Semi-structured qualitative interviews	Reflexive thematic analysis	United Kingdom
2019	Sun et al. [36]	To explore perceptions of treatment outcomes among Chinese families with adolescents with AN	6 Parents (4 mothers and 2 fathers)	Semi-structured interviews	Grounded theory	China
2020	Sibeoni et al. [37]	To explore the experience of inpatient treatment therapeutic alliance among adolescents with AN, parents, and psychiatrists	18 Parents (14 mothers and 4 fathers) and 15 females (aged 13–17)	Semi-structured interviews	Thematic analysis	France
2022	Sourlier et al. [38]	To explore the experiences of parents and anorexia nervosa patients after reorganization of care caused by COVID-19	6 Mothers and 6 fathers of 6 patients (aged 10–18)	Semi-structured interviews	Inductive thematic analysis	France
2022	Giombini et al. [39]	To qualitatively explore the views of young people and parents towards individual cognition remediation therapy with randomised controlled trial	47 Parents of 75 females and 5 males aged 10–18 years old	Open-question questionnaire	Thematic analysis, summative content analysis, and coding	UK
2021	Lockersten et al. [40]	To qualitatively explore parents' experiences with the transition process from child and adolescent to adult mental health services for AN patients	12 Parents (9 mothers and 3 fathers) of 10 adolescents aged 15 on average	In-depth, semi-structured interview	STC (systematic text condensation)	Norway
2021	Nilsen et al. [41]	Thematic analysis of the reflections on family-based treatment programs for adolescent nervosa by family members'	14 Parents of 8 patients (aged 12–18 at admission)	Semi-structured interviews	IPA	Norway
2020	Williams et al. [42]	Examine parents' experiences of family-based interventions for the treatment of adolescents with AN	9 Parents (6 mothers and 3 fathers) of 7 patients (aged 12–18 at admission)	Semi-structured interviews	Thematic analysis (QSR-NVivo9)	Australia
2022	Whitney et al. [43]	Explore mothers' caregiving experiences prior to diagnosis through post inpatient treatment	10 Mothers of 10 sons (aged 10–17 at diagnosis)	Semi-structured interviews	CQR methods	US
2022	Thibault et al. [44]	Thematic analysis of the experiences of parents that are involved in family-based treatment	6 Parents (6 mothers) of 1 male and 5 females (aged 11–17)	Semi-structured interviews	Thematic analysis + NVivo 12	Canada

### Quality assessment

Evaluation of studies using CASP showed 22 out of the 25 studies met at least 8 (out of 10) criteria. All studies but one stated receiving ethical approval but did not clearly state how they dealt with issues around informed consent or confidentiality. Author reflexivity, which considers how a researcher critically examines their own role for potential basis in the research and how they have dealt with this, was only fully met by six studies.

A summary of the analysis using the CASP 10 questions is shown in Table 3. A detailed table outlining the individual assessment of each article is included in Additional file 1: Appendix A.

**Table 3:** Critical appraisal skills program (CASP) quality assessment summary

Criteria	Definition	Met criterion	Partially met	Did not meet	Can’t tell
Aim	Clear statement of aim of research	25	0	0	0
Method	Appropriate use of qualitative methodology	25	0	0	0
Research design	Justification of research design	22	3	0	0
Sampling	Recruitment appropriate to research aim (including participant selection)	22	3	1	0
Data collection	Appropriate data collection and description of collection methods	24	1	0	0
Reflexivity	Critically examination of researcher’s role and potential for bias	6	7	12	0
Ethical Issues	Approval from ethics committee including appropriate discussion with participants regarding confidentiality/ informed consent	14	6	4	1
Data Analysis	Rigorous data analysis and description of process	22	3	0	0
Findings	Clear statement of findings with discussion of credibility of findings	25	0	0	0
Research value	Contribution to existing knowledge and transferability of findings	25	0	0	0

## Thematic analysis

Thematic analysis yielded four central themes and twelve sub-themes from the studies. Themes described the journey embarked on by parents with their adolescent’s AN and suggested helpful aspects of care and areas for improvement.

A detailed list of quotes illustrating the major themes and sub-themes is presented in Table 4.

**Table 4:** Quotations from participants in primary studies illustrating each theme

Theme	Sub theme	Quotes from studies
1. Understanding the child and the disease	Initial reaction to diagnosis	“Oh my God, she is really ill” [28] “I was appalled by her skin and bones appearance” [20] “The initial diagnosis came out of the blue” [31] “…I suppose really would be the initial stage of shock, we found it very hard” “… it’s the worst thing ever and its happening under your nose…” [27] “I thought it was for attention. I thought it was a phase. I had no idea how serious.” [43]
	Guilt/self-blame	“I didn’t see the beginning of anorexia for a while” [29] “I felt I should have picked up on it earlier” [23] “I felt this happened to other people that weren’t as good parents as we were” [45] “I should have been more watchful” “did we not pay enough attention?” [33] “I was really busy going to university and as well as work…when I realised that she wasn’t eating that was just a shock; I just felt guilt” [42] “I wonder, did I do anything wrong? Did I show that [pause] well I probably showed that I didn’t like my body” [44]
	Helplessness	“We’d failed as parents” [33] “I felt helpless” [45] “…I kind of think that nothing I do helps really” [26] “I was…often crying a lot..” [28] “…she needs help in a way that she doesn’t know she needs…There is no meaning” [43] “There was nothing we could do after trying 10 different types of treatments” [36] “A mother’s heart bleeds when I see how she treats her sister, but what can I do?” [40]
	Loneliness and stigma	“It was just me and her…” [26] “It is lonely… friends were frightened to come…” [28] “..we’d go out for meals with people.. that’s stopped because.. she found it difficult” [23] “If he had cancer or a surgery, my friends would have called” [43] “My brother, I have the intention to talk to him about it during the holidays, but he doesn’t know about it yet” [44]
2. Experience of services and treatment modalities	Help seeking	“… the initial contact with the GP is not great…” “They need to understand it’s not just about the weight…” [31] “If I were to express any misgivings, it will be about the time it took to get a referral from her GP to CAMHS…” [30] “I went to her doctor who was an old doctor…..and…. he just totally dismissed her” [27] “He said that I was making too much fuss and you know she was a teenager and she was fine…” [28] “…well you can’t be anorexic because you’re actually eating” [21] “I’m still cross that the GP hadn’t confirmed it early on…” [23]
	Experience of treatment modalities: *Positive experiences*	On FBT: “We were a joint force” “It developed an openness that hadn’t been there before” “…as a family we’ve grown a lot closer…” [45] “I think it has been good for both fathers and mothers…” [29] “…I think the kids found it quite useful because they could say what they’d been bottling up for a long time…” [21] “…we can discuss those unhappy things. I feel good about it” [25] “Some things you can only talk about with others who experienced it” [31] “Attending the parenting courses was very helpful…” (Nilsen 2021) On home therapy: “I felt energised…” [26] On MFT: “…if it came from my child, I would not have understood. I understood better through the words of another young person” [34] “…seeing…[what] people had triumphed over or their views being slightly different was quite…interesting and helpful” [35]
	Experience of treatment modalities: *Negative experiences*	On FBT: “The focus seems to be all on the food aspect” [21] “Really what you need is someone who sees the whole person…” [30] “…they didn’t appear to be doing very much or to be talking about the whys and wherefores” [24] “When things aren’t going well, you kind of blame yourself” [33] “When we were on our own… we could have been more honest” [21] “I really don’t know where you could find family therapists that specialize in treating anorexia in Hong Kong…such kind of support is totally unavailable” [36] On in-patient treatment: “there was a lot of moping around….” [21] “How thin must one become to receive treatment? Actually, she lost weight just to meet their BMI criteria” [40]
	Compulsory treatment/control	“…I think if their life is at risk… someone has to make the decision…” [22] “…She isn’t strong enough to make a decision about her” [21] “If you give treatment in early stages… you are prolonging the time they can get well” [21] “I think if someone is going to starve themselves to the point where they are dying… you must be allowed to die quietly, kindly” [22] “I think probably it was good for her to know that somebody else was in control…” [23] “…you don’t have much choice, because in any case you don’t know what to do any more.” [37]
3. The role of professionals	Helpful attributes: availability	“She (paediatrician) was there at our beck and call….. she said, ‘I’ll do whatever you need” [21]
	Support	“…. He (psychiatrist) just kind of saved us in a way” [21] “…the key to her improvement…she trusted her therapist completely…” [26]
	Sensitivity and competence	“She (therapist) would calm us down…” [31] “When things were really bad (the professional)… was wonderful” [23]
	Unhelpful attributes: lack of feedback and poor communication	“…something would happen and I’d think, how do I approach it?…” [21] “…there wasn’t any other feedback other than ‘oh well that’s difficult’…” [24] “…in hospital no one spoke to us…” [24] “…they had never told me that they had a treatment plan…” [36]
	Poor communication between professionals	“…sometimes nurses don’t know about a decision the consultant had made…” [21] “If I try to consult all types of healthcare professionals…I would have to explain everything from beginning when I meet each of them…what they said…were inconsistent” [36]
	Rigidity among professionals	“She (therapist) had a framework she wanted to fit us in…” [21]
	Being undermined	“…my husband and I found the psychiatrist condescending…” [24] “…we were scolded by the psychiatrist…He thought that we didn’t care about our daughter” [36]
	Negative attitudes	“I do feel that many clinicians (not necessarily ED specialists) believe that parents cause eating disorders” [30] “I felt very judged… at a time in my life when the core thing I needed was support” [31] “Even treatment professionals question whether I caused this disorder” [43]
	Limited knowledge	“…nutritionists were quite unfamiliar with the disease…They only asked her to eat because she looked skinny” [36] “I [still] couldn’t get any local, knowledgeable, experienced care for my son…I think that our medical community could use real serious [education]” [43]
4. The experience of recovery	Changes in the adolescent	“…she talked about wanting to get a job…” [29] “She has matured …and now wants to get better” [27] “…She is more flexible and open about changes and new activities” [39] “She’s been very relaxed over the past week… whereas she wasn’t at all before, she was self-centred” [38]
	Uncertainty about the future	“It’s all unknown” “I hope she’s ok”[21] “What on earth is going to happen next with this..?”[24] “…..not knowing that is the hardest of it…”[33] “Like if he goes out in the worl, will he be prepared? Will he be able to handle himself? We keep thinking, ‘Is he going to grow out of it?’” [43] “I thought the transition was a bit sudden, you know, with her coming home and having to return to school and then having to cope with everything.” [38]
	Experience of relapse	“…deteriorating before our eyes…” “I find it quite depressing…” [21] “…taking one step forward and then three back…” [32] “… to accept relapses was difficult. To accept that it didn’t go according to the pace I had hoped…” [44]

### Understanding the child and the disease

Most studies described parents’ initial struggle with coming to terms with their child’s diagnosis and understanding the meaning or the cause of the disease. Parents report a slow recognition of the problem after initially attributing changes in their adolescent’s behaviour to a passing phase of adolescence or ignoring the signs of AN as a way of avoiding confrontation at meal times [21, 23, 42, 43]. Three studies described confrontations between the adolescent and a parent, which were difficult in each instance [20, 22, 28].

Five sub-themes were identified as recurring in the majority of studies.

#### Initial reactions to diagnosis

Seven studies report parents being taken aback by the severity of the symptoms as their child further deteriorated, which brought about a realisation of how ill their child was [20, 23, 27, 29, 30, 42, 43]. The initial reaction was predominated by denial and shock at discovering their child had an ED, which they never thought would happen in their own home.

#### Feelings of guilt and self-blame

Seven studies [20, 28, 30, 32, 42–44] reported on parents’ struggle with guilt and self-blame. Parents often blamed themselves for not recognising the illness earlier, not seeking help, or even believing they were ‘causing the illness’[43]. One father particularly felt he had failed in his role as ‘a protector’ of the family [32]. Parents blamed themselves for contributing to their child’s illness by not watching them closely or by dieting in the past [30, 32, 38, 44]. Guilt occurred at two time periods – at diagnosis and when the required weight gain was not achieved or maintained during therapy.

#### Anxiety and desperation

Anxiety and desperation during the treatment process featured in six studies [22, 23, 28, 30, 38, 45]; parents were worried about knowing too little about the disease and not knowing the treatment process, which some found unbearable [23]. Anxiety was heightened by self-blame [30].

#### Feelings of helplessness

Eight studies [20, 22, 32, 36, 40, 42–44] reported on the parents’ account of feelings of helplessness or powerlessness, which further heightened their sense of failure. Feelings of hopelessness may lessen the effectiveness of a parent’s role in a child’s treatment, as some parents may begin to believe that there is nothing that can be done to improve the situation.

#### Loneliness and stigma

Four studies [22, 30, 43, 44] reported parents struggling with loneliness. As their responsibility for caring for their child increased, they felt further isolated, stigmatised, and cut off from their friends. Parents felt that stigma around mental illness and EDs contributed to a lack of support and increased blame from peers [43].

### Experience with services and treatment modalities

Studies varied in their reporting of service experience by parents, depending on the mode of therapy their participant’s child was involved in at the time of the study.

Recurring themes were found as the initial process of seeking help, as well as the experience of different treatment modalities and settings. A third sub-theme that was featured less often concerned compulsory treatment and control.

#### Help-seeking

Most parents describe actively seeking help with the progressive manifestation of their child’s AN after trying to resolve issues within the family. The first point of contact for many was often their family doctor.

Six studies [22, 24, 28, 30, 31, 45] reported that an initial barrier in help-seeking was a perceived knowledge gap by their doctor in the area of EDs, as there was an inability to provide required information or information on treatment options. There was a misconception that the adolescent was ‘not ill enough’ and a subsequent dismissive reaction [22, 24, 45], with some parents presenting multiple times before their concerns were taken seriously, although this did not necessarily mean that the doctor was unwilling to assist.

#### Experience with treatment modalities

##### Family-based/multi-family treatment (FBT/MFT)

Ten studies focused mainly on the experience of family-based treatments [23, 25, 29, 32–35, 41, 42, 44], including a FAP [29].

Overall, FBT was seen as positive by parents, being of a practical nature and helping them be more active in their child’s treatment. FBT helped the family to engage in difficult conversations that enabled them to also deal with guilt and fostered closeness between family members.

Although viewed relatively less favorably than FBT, MFT was viewed positively by some parents who felt meeting with other parents in a similar situation made them feel less lonely, with some relief from their shame and guilt [28, 34, 35]. MFT had a positive effect on parental disputes, leading to more shared responsibility and greater ability to vocalise their feelings [23, 29, 31–33]. They felt empowered, more able to deal with the illness, and had a better understanding of their child [35, 41].

The FAP, which allowed families to reside within the hospital during an acute treatment phase, was positively viewed by parents because of the proximity to support and professionals, especially ‘after hours,’ thus creating a sense of safety [29].

Parent groups offered by some services offered valuable opportunities for parents to connect [24, 28, 41]. Peer support often helped parents feel empowered and validated, while also lessening their feelings of shame and guilt.

Some parents viewed sibling involvement as positive in improving the relationship between siblings by providing an environment for expressing themselves. However, some parents whose children had a shorter duration of illness preferred not involving siblings [23]. Other studies highlighted negative aspects of experiences with FBT/MFT, though parents overall had more concerns about inpatient care compared to FBT/MFT. Five studies [24, 26, 32, 33, 40] highlighted a general concern regarding an imbalance between physical and psychological aspects of care, with a focus on patients’ weight and neglect of psychological struggles, and subsequently advocated for a holistic approach.

With regard to MFT, some families felt that the heterogeneity of groups made it difficult to address family-specific issues [34]. Others were worried that the group setting worsened symptoms by introducing sources of comparison with other children in therapy or by shifting the focus away from the individual, contributing to feelings of worthlessness [35, 42].

Parents also reported distress with taking over the responsibility for their child’s feeding during the first phase of FBT when they felt unable to take up this role and felt guilt when they could not achieve this task.

Some parents expressed frustration with always being in a session with their child [26, 45] and felt there should have been opportunities ‘where we were on our own’ [45] in order to fully express themselves and also felt that sometimes the child wanted to be by themselves in the room with the therapist.

##### Home treatment

One study reported on home treatment—an intensive support program that focuses on mothers as the primary caregivers and addresses the adolescent’s needs in the home and family environment when they failed outpatient therapy [22]. Parents experienced containment, provision of hope, an opportunity to find strength, and a reduction in the burden of care [41].

On the negative side, parents reported fear and anxiety about the future when home therapy ended as it could only be provided for a certain period of time; some parents experienced anxiety from being ‘observed’ [35, 44].

##### Inpatient treatment

Two studies talked about parents’ experience of inpatient treatment as inappropriate as it was in a general ward. Nurses spent little time with the patient, and even in a specialist ward, a shortage of staff meant that optimum care was not provided. Feelings of guilt were exacerbated by not being with their child constantly due to other commitments, travel costs, and being torn between spending time with their child in the hospital and their other children [21, 36].

#### Compulsory treatment/control

One study described the parents’ views of compulsory treatment [22] as a conflicting period for patients, while another study mentioned ‘control’ as a sub-theme [23]. Their understanding of their child’s vulnerability and poor decision-making skills during the illness meant they trusted the professionals to assist them in decision-making. Parents emphasized that part of their role was ‘taking control,’ even if it meant forcing their child to attend appointments.

However, they were conflicted between acknowledging that compulsory treatment was justified and wanting their child to be treated with dignity (their consent), as exemplified by the comment, ‘…I had two conflicting opinions’ [22]. They felt that compulsory treatment did not help with engaging the child with the psychological treatment that was required to ultimately battle the illness. There was disagreement among parents as to the right time to implement compulsory treatment – some parents felt it should happen early in the disease process, while others felt it should be left until there were life-threatening circumstances.

### The role of professionals

This was a recurring theme found in at least twelve of the studies.

Parents had expectations of professionals treating their children for physical and psychological support. Eight studies [20, 25, 27, 29, 33, 36, 40, 41] especially described parents requiring a relationship with professionals to involve them, support them, and keep them informed. Information was sought about AN with clear guidance and regular feedback. Parents sought emotional support and a link to parental support groups [23, 25, 27, 29].

They also described a relief in finding professionals who specialised in the treatment of AN, although in some cases, there were delays in finding this help and incurred costs of travelling [24, 32]. Some parents felt that therapists who had experienced an ED in the past understood them better [20].

Two sub-themes were identified in most studies as parents describing what they found helpful and unhelpful in dealing with professionals.

#### Helpful attributes

Availability, competence, and positive attitudes like empathy, sensitivity, respectfulness, friendliness, and trustworthiness were especially highlighted in six studies [20, 24, 26, 31, 33, 36] and were deeply appreciated by parents. There was a high sense of gratitude to professionals who were readily available, with a parent referring to their psychiatrist as ‘some kind of saviour’ [24]. Parents resented being excluded from the adolescent’s treatment and found ‘being listened to’ very helpful.

#### Unhelpful attributes

Nine studies [24, 25, 27, 28, 31, 32, 36, 40, 43] highlighted attributes that parents found very unhelpful when dealing with professionals involved in their child’s care.

Five studies [24, 27, 28, 36, 40] reported on a lack of feedback from clinicians, which made them unclear about their child’s prognosis during therapy. Parents reported frustration with poor communication between professionals and parents, as well as poor communication between professionals themselves [24, 25]; they felt that while their child was involved with many professionals, the professionals were not communicating well with each other.

Four studies reported the experiences of parents who had previously seen ‘professionals who lacked knowledge about EDs’ [30, 31, 36, 43]. Parents felt that doctors, nutritionists, social workers, and other members of the medical community who were unfamiliar with AN negatively impacted the diagnosis and treatment of their child [36, 43].

Parents reported negative attitudes where they felt like they were being blamed or judged [43] for their child’s illness and experienced rudeness from professionals, with some undermining their role as parents and excluding them in decision-making [24, 25, 27, 28, 31, 36]. Some professionals were rigid in their approach and did not focus on their child as an individual [24].

### The experience of recovery

This theme was recurrent in eleven studies [21, 24, 27, 29, 32, 33, 38, 39, 42–44]. Parents recognised recovery when it occurred as an ongoing process. Hope was essential [24, 27, 32]. Communication (e.g., regarding follow-up, the need for ongoing support, and post-discharge planning) was highlighted in seven studies [24–27, 30, 32, 43]. Three sub-themes were developed in recovery from AN.

#### Changes in the adolescent

Six studies reported parents observing an overall positive outlook with recovery, personality change, better engagement with the family, and increased confidence with recovery [23, 25, 30, 33, 38, 39].

Parents noted that their adolescents had matured during the treatment process and wanted to remain well [29].

#### Uncertainty about the future

Five studies [21, 24, 33, 38, 43] reported the parents’ struggle with uncertainty during the process of AN treatment for their child. Although most parents had a more positive outlook as their child progressed through treatment with a reduction in AN symptoms, there was a lot of uncertainty during the recovery period and difficulty in predicting the future while fearing that AN ‘might return.’ This uncertainty was exacerbated if professionals had not adequately communicated the lengthy process of treatment and recovery to parents. Some parents voiced a sense of frustration with the lack of feedback and guidance at the end of home therapy [26].

#### Experience of relapse

Four studies reported an experience with relapse [24, 26, 28, 44]. Parents experienced substantial anguish when relapse occurred, especially after inpatient therapy [24] or home therapy [26]. It was agonising watching their child deteriorate after they had made some progress, and parents struggled to find support during this time from professionals. Some even found it depressing [24].

## Discussion

This systematic review and metasynthesis provides insights into parents’ significant role in the treatment of AN for their adolescent children starting from the period when they face the diagnosis to working through the challenges of treatment and the recovery process. Our results show an emphasis on parents and the importance of developing a therapeutic relationship between parents and professionals for treatment success.

In our first theme, we found across studies the extreme distress, guilt, self-blame, and anxiety faced by parents at the time their child is diagnosed with AN and during the course of the disease. This is consistent with studies that have reported high levels of psychological distress and expressed emotion in carers of people with EDs [46, 47]. Clinicians may need to address this first by providing reassurance, prognostic information, and managing the expectations of parents at the start of the illness. This is especially important, given that responsibility for re-feeding their adolescent is handed to them early in treatment when these struggles may be at their highest levels.

Given the high burden of care with AN, the importance of the role of parental involvement in treatment has been well established, with the best outcomes being achieved when treatment providers and families work together [5, 48]. Adolescents have also reported a positive effect on treatment with the participation of their parents [49].

While parents acknowledge the helpful input of professionals in their child’s treatment, they report that treatment does not necessarily consider their adolescents’ psychological and social functioning. This may signify a difference in expectations between parents and professionals on the definition of recovery. A review describing the intersecting views of adolescents, parents, and clinicians in the treatment of AN reported a dissonance in treatment targets between parents, adolescents, and professionals [5]. Several studies have shown that recovery should not be defined only by behavioural and physical criteria but must take the individual’s psychological functioning into account in order to sustain recovery [50, 51]. A normalisation of weight among recovered adolescents with the persistence of anorectic cognitions and food restrictions presents a high risk of relapse [52].

While the evidence suggests that early intervention in children with AN leads to better recovery rates [53], our study showed that many parents report a delay in diagnosis and appropriate referral. The difficulty in accessing appropriate services reported in earlier studies persisted in more recent studies. A knowledge gap in the symptoms of AN by primary care physicians has also been reported in previous studies [54, 55].

Our findings suggest a number of areas of improvement for clinicians in caring for adolescents with AN in collaboration with parents. Our findings highlight the importance of tailoring treatment to specific family and patient needs rather than utilizing a ‘one size fits all’ treatment approach.

This review provides reports on parental perceptions of professionals’ attitudes and what attributes they found helpful or unhelpful. In line with previous studies [20, 47], parents continue to report areas of unmet needs with regard to professional support.

Parents provided information regarding the usefulness of different services and how these services can provide better assistance to families. This is described in the second and third central themes of our results. Differences in perception and expectations should be managed and incorporated into the holistic management of their child’s illness. While FBT is a well-established treatment for adolescent AN, current challenges exist. Studies have shown that the current manualised version of FBT is not suitable for families with highly expressed emotions and parental criticism [45]. Some adolescents also continue to experience psychological distress after completing therapy even though they might be fully ‘weight restored.’ Our results highlight some barriers to treatment and recovery and underscore a need for continued dialogue between parents and professionals to strengthen therapeutic relationships. Parents strongly advocated for support groups involving other parents with similar experiences.

### Strengths and limitations

This study integrates views from 357 parents, focusing primarily on their views about the treatment of their adolescents with AN. We used a rigorous methodology [13], analysing 25 articles, all of which were published in peer-reviewed journals. This is the first qualitative metasynthesis to exclusively capture the views of parents and cuts across perspectives from different services at different stages of their child’s illness, thereby providing diverse treatment perspectives.

In many studies, treatment was carried out over a long period so experiences may have differed depending on the child’s stage of treatment There is variability in when the data was collected, which may have further affected results. Participants were chosen based on their ‘willingness’ to partake in the studies; it is possible that this may have led to selection bias. Only a few studies stated reasons for a decline in participation.

The generalisability of results of this metasynthesis may be limited, given that most of the studies were conducted in Western countries with a lack of views from racial and ethnic minorities and lower socio-economic classes.

There was also an underrepresentation of parents of males, with only six studies mentioning sons with AN [31, 33, 34, 39, 43, 44]. Fathers are also under-represented, with some studies having only interviewed mothers. Parents of boys and fathers of patients may have different views that also need to be explored.

### Implications for future research and practice

This synthesis provides some insight into barriers to treatment and ways clinicians can improve the experience for parents. By highlighting the negative experiences parents have had with accessing or navigating care, we highlight a need for healthcare systems and governments to improve the accessibility of services for EDs. In addition, this study highlights the role of the professional, indicating the importance of adequately training medical providers to manage EDs. Further research is also needed to elicit the views of fathers, parents of boys with AN, and ethnic minorities.

The findings in this study also invite further research in the area of MFT, which may work better for families who struggle on their own. The home therapy model [26] and FAP [29] also require further investigation of models with more comprehensive AN treatment that not only ensures weight gain but lays emphasis on a more holistic approach that addresses the psychological impact of AN on the child’s identity and development.

Finally, an important finding is the gap in transparent reporting of ethical issues and reflexivity—which was not addressed in a sizeable portion of the qualitative studies. This has implications for future researchers and highlights a need for training in this area in order to ensure good quality research.

## Conclusion

The studies reviewed highlight the importance of qualitative methods in clinical research and underscore the importance of parental involvement in the treatment and recovery of AN in adolescence. Building strong therapeutic relationships strengthens the achievement of optimal treatment goals, supports recovery and prevention of relapse, and informs future service delivery.

### Supplementary Information

**Additional file 1: Appendix A** Evaluation of study quality of included studies using the Critical Appraisal Skill Program (CASP). Evaluation of study quality of included studies using the Critical Appraisal Skill Program (CASP).

## Data Availability

The data that support the findings of this study are available from the first author upon reasonable request.

## Abbreviations

AN: Anorexia nervosa
CASP: Critical appraisal skills program
ED: Eating disorder
FAP: Family admissions program
FBT: Family-based treatment
FBT/MFT: Family-based treatment/multi-family treatment
