# Supplementary material for: Parents’ perspectives of anorexia nervosa treatment in adolescents: a systematic review and metasynthesis of qualitative data
Source: J Eat Disord. 2023 Oct 30;11:193. doi: 10.1186/s40337-023-00910-z (PMC10617236; doi:10.1186/s40337-023-00910-z)
Supplement: Supplementary file 1 — Additional file 1: Appendix A Evaluation of study quality of included studies using the Critical Appraisal Skill Program (CASP). Evaluation of study quality of included studies using the Critical Appraisal Skill Program (CASP). [file 40337_2023_910_MOESM1_ESM.docx]

**APPENDIX A**

Evaluation of study quality of included studies using the Critical Appraisal Skill Program (CASP).

Y: Met criterion; C: Can’t Tell; P: Partially met criterion; N: Did not meet criterion

| References | Aims | Methods | Research  Design | Sampling | Data  collection | Reflexivity | Ethical  Issues | Data  Analysis | Statement of Findings | Value of  Research | Total (out of 10) |
| --- | --- | --- | --- | --- | --- | --- | --- | --- | --- | --- | --- |
| Sharkey-Orgnero (1999) [17] | **Y** | **Y** | **Y** | **Y** | **Y** | **N** | **N** | **Y** | **Y** | **Y** | **8** |
| Tan et al (2003) [19] | **Y** | **Y** | **Y** | **Y** | **Y** | **N** | **C** | **Y** | **Y** | **Y** | **8** |
| Cottee-Lane et al (2004) [20] | **Y** | **Y** | **Y** | **Y** | **Y** | **N** | **N** | **Y** | **Y** | **Y** | **8** |
| Tierney (2005) [18] | **Y** | **Y** | **Y** | **Y** | **Y** | **Y** | **P** | **Y** | **Y** | **Y** | **9** |
| Honey et al (2007) [27] | **Y** | **Y** | **Y** | **Y** | **Y** | **N** | **N** | **Y** | **Y** | **Y** | **8** |
| Ma (2008) [30] | **Y** | **Y** | **Y** | **N** | **P** | **N** | **N** | **P** | **Y** | **Y** | **5** |
| Bezance and Holliday (2014) [31] | **Y** | **Y** | **Y** | **P** | **Y** | **Y** | **P** | **Y** | **Y** | **Y** | **8** |
| McCormack and McCann (2015) [24] | **Y** | **Y** | **Y** | **Y** | **Y** | **N** | **Y** | **Y** | **Y** | **Y** | **9** |
| Engman-Bredvik et al. (2016) [21] | **Y** | **Y** | **Y** | **Y** | **Y** | **N** | **Y** | **Y** | **Y** | **Y** | **8** |
| Fink et al (2017) [22] | **Y** | **Y** | **Y** | **Y** | **Y** | **Y** | **P** | **Y** | **Y** | **Y** | **9** |
| Mitrofan et al (2019) [23] | **Y** | **Y** | **Y** | **Y** | **Y** | **P** | **Y** | **Y** | **Y** | **Y** | **9** |
| McArdle (2019) [28] | **Y** | **Y** | **Y** | **Y** | **Y** | **N** | **Y** | **Y** | **Y** | **Y** | **9** |
| Medway et al (2019) [25] | **Y** | **Y** | **Y** | **Y** | **Y** | **N** | **P** | **Y** | **Y** | **Y** | **9** |
| Wufong et al (2019) [29] | **Y** | **Y** | **Y** | **Y** | **Y** | **N** | **P** | **Y** | **Y** | **Y** | **8** |
| Baumas et al (2021) | **Y** | **Y** | **Y** | **Y** | **Y** | **P** | **Y** | **P** | **Y** | **Y** | **8** |
| Baudinet et al (2023) | **Y** | **Y** | **P** | **Y** | **Y** | **Y** | **Y** | **Y** | **Y** | **Y** | **9** |
| Sun et al (2019) | **Y** | **Y** | **Y** | **Y** | **Y** | **Y** | **Y** | **Y** | **Y** | **Y** | **10** |
| Sibeoni et al (2020) | **Y** | **Y** | **P** | **Y** | **Y** | **N** | **Y** | **P** | **Y** | **Y** | **7** |
| Sourlier et al (2022) | **Y** | **Y** | **Y** | **P** | **Y** | **P** | **Y** | **Y** | **Y** | **Y** | **8** |
| Giombini et al (2022) | **Y** | **Y** | **Y** | **P** | **Y** | **P** | **Y** | **Y** | **Y** | **Y** | **8** |
| Lockersten et al (2021) | **Y** | **Y** | **Y** | **Y** | **Y** | **P** | **P** | **Y** | **Y** | **Y** | **8** |
| Nilsen et al (2021) | **Y** | **Y** | **Y** | **Y** | **Y** | **P** | **Y** | **Y** | **Y** | **Y** | **9** |
| Thibault et al (2022) | **Y** | **Y** | **Y** | **Y** | **Y** | **P** | **Y** | **Y** | **Y** | **Y** | **9** |
| Williams et al (2020) | **Y** | **Y** | **P** | **Y** | **Y** | **N** | **Y** | **Y** | **Y** | **Y** | **8** |
| Whitney et al (2023) | **Y** | **Y** | **Y** | **Y** | **Y** | **Y** | **Y** | **Y** | **Y** | **Y** | **10** |
